# Effects of exercise therapy on balance, functional performance, and fall-related outcomes in community-dwelling older adults with chronic conditions: a systematic review and meta-analysis

**DOI:** 10.3389/fimmu.2026.1918701

**Published:** 2026-10-20

**Authors:** Xiaofeng Cheng, Jinsong Luo, Beihai Ge, Deng Wang, Renming Liu, Xianhui Liao

**Affiliations:** 1 Department of Infectious Diseases, Shangyu People's Hospital of Shaoxing, Shaoxing University, Shaoxing, China; 2 Wuchang University of Technology, Wuhan, Hubei, China; 3 Rugao People's Hospital Affiliated to Kangda College of Nanjing Medical University, Nantong, China; 4 School of Physical Education, Guangzhou University of Sport, Guangzhou, China; 5 Monash Neuromodulation Research Unit, Department of Physiotherapy, School of Primary and Allied Health Care, Monash University, Melbourne, VIC, Australia; 6 Department of Physiotherapy, School of Primary and Allied Health Care, Monash University, Melbourne, VIC, Australia

**Keywords:** balance, community-dwelling, exercise therapy, fear of falling, functional performance, older adults, systematic review and meta-analysis

## Abstract

**Background:**

Community residents with chronic diseases face a higher risk of falls, functional decline, and loss of independence, constituting a significant public health burden. Exercise therapy is widely recommended; however, its efficacy in improving balance, functional performance, and fall-related outcomes remains controversial.

**Objective:**

To systematically evaluate and quantify the effects of exercise therapy on balance, functional performance, and fall-related outcomes in community-dwelling older adults with chronic conditions.

**Methods:**

This study conducted a systematic review and meta-analysis of randomized controlled trials. PubMed, Cochrane Library, Embase, CINAHL, and Web of Science databases were searched, and studies published up to 8 March 2026 were included. Participants were community-dwelling adults with chronic diseases who received exercise therapy. A random-effects model was used to calculate the pooled effect size. Outcome measures included balance, functional performance, and fall-related indicators. Subgroup analyses were conducted according to chronic condition categories and exercise modalities to explore potential sources of heterogeneity.

**Results:**

A total of 14 randomized controlled trials involving 857 participants were included in the meta-analysis. Exercise therapy significantly improved multiple objective indicators of balance and functional performance in community-dwelling older adults with chronic conditions. Significant improvements were observed in the One-Leg Stance (OLS) (WMD = 5.45, 95% CI: 1.45 to 9.44), In addition, exercise therapy significantly reduced Timed Up and Go (TUG) (WMD = −0.76, 95% CI: -1.50 to -0.02), indicating improved functional mobility and dynamic balance. Subgroup analyses suggested that improvements in TUG performance varied according to chronic condition categories and exercise modalities, with greater benefits observed among participants with musculoskeletal-related chronic conditions and those receiving balance training.

**Conclusions:**

Exercise therapy may improve selected measures of balance and functional mobility in community-dwelling older adults with chronic conditions, although the certainty and clinical importance of these effects remain uncertain. Effects on falls and fear of falling were not established in this review. Further high-quality studies are needed to determine the optimal exercise characteristics for specific chronic condition populations.

**Systematic review registration:**

https://www.crd.york.ac.uk/PROSPERO/, CRD420261390054.

## 1 Introduction

Aging population has become a major challenge to global public health, with a significant increase in the prevalence of chronic diseases among the elderly. As life expectancy increases, older adults suffer from multiple diseases, including cardiovascular (1), metabolic (2), musculoskeletal (3), and neurological disorders (4). Among them, older adults aging at home constitute a crucial yet vulnerable group, struggling to maintain independent living despite deteriorating health. This group is more susceptible to functional decline, balance disorders, and limited mobility, all of which increase the risk of falls (5, 6).

Falls are a leading cause of injury, disability, and death among older adults, placing a heavy burden on individuals and society (7). Beyond acute physical consequences such as fractures and traumatic brain injury, falls often lead to persistent functional decline, residency in nursing homes, and loss of independent living abilities. More importantly, the psychological sequelae of falls—including fear of falling and decreased self-efficacy—further restrict older adults’ activity, accelerating physical decline and exacerbating the vicious cycle of decline (7). From a systemic perspective, fall incidents lead to increased utilization and expenditure of healthcare resources, highlighting the urgency of developing effective prevention strategies (8). Therefore, maintaining functional capacity in older adults while reducing fall risk has become a core objective in the management of elderly patients with chronic diseases.

Exercise therapy is widely considered a cornerstone nonpharmacological intervention for addressing these challenges (9). Evidence suggests that various exercise modalities, including balance training, resistance training, aerobic training, and multi-component training, can improve neuromuscular function, muscle strength, coordination, and postural control, thus targeting key physiological determinants of falls (10–12). Simultaneously, exercise can also influence behavioral and psychological aspects, such as self-confidence and perceived stability, factors crucial for functional performance in real life (13). These multidimensional effects make exercise therapy a theoretically reliable intervention for fall prevention and functional maintenance (9, 11).

limitation of the existing literature lies in its fragmented assessment of outcome measures. Many studies prioritize isolated endpoints, such as balance or fall incidence, while neglecting the multidimensional nature of functional abilities. Objective measures (standardized balance tests or functional performance assessments) and subjective outcome measures (perceived fall risk, self-confidence, or fear of falling) are often assessed separately (14). Notably, emerging evidence suggests that improvements in objective physical function do not always translate into corresponding increases in self-efficacy or perceived safety, this inconsistency highlights a crucial gap, indicating that improvements in physical function alone may not be sufficient to produce significant changes in behavior and actual fall risk (15).

Moreover, fall-related outcomes are inherently complex, encompassing not only fall occurrence but also fall rates, recurrent falls, fall-related injuries, and psychosocial factors. Ignoring this complexity can lead to an incomplete understanding of intervention effects and even misleading conclusions. To date, few studies have comprehensively integrated these diverse outcomes into a unified analytical framework, and even fewer have quantitatively synthesized different trials.

In summary, these limitations underscore the necessity for a rigorous and comprehensive analysis of existing evidence. Therefore, this systematic review and meta-analysis aim to assess and quantify the impact of exercise therapy on balance, functional performance, and fall-related outcomes in community-dwelling older adults with chronic diseases. By integrating data from randomized controlled trials and examining objective and subjective endpoints, this study aims to elucidate the multidimensional effects of exercise therapy, explore sources of heterogeneity, and provide relevant evidence.

## 2 Methods

This systematic review and meta-analysis were conducted in accordance with the PRISMA (Preferred Reporting Items for Systematic Reviews and Meta-Analyses) guidelines (16). The review protocol was prospectively developed to ensure methodological transparency and reproducibility. The protocol was registered in PROSPERO (Registration CRD420261390054).

### 2.1 Eligibility criteria

Studies must meet the following criteria for inclusion: (1) Participants must be community-dwelling older adults aged ≥60 years with one or more age-related chronic diseases that lead to functional decline, balance disorders, limited mobility, or increased risk of falls; (2) A randomized controlled trial (RCT) design must be used; (3) Interventions must include exercise therapy, such as general physical activity, resistance training, balance training, aerobic exercise, Tai Chi, Baduanjin, or multi-component exercise therapy; (4) Control groups must include routine care, no intervention, or a non-exercise control group; (5) Outcome measures must include at least one balance, functional performance, or fall-related outcome measure (fall incidence, fear of falling); (6) Articles must be published in English. Chronic conditions were defined as long-term health conditions associated with persistent functional limitations and requiring ongoing management. Eligible chronic conditions included musculoskeletal-related conditions (e.g., osteoporosis and sarcopenia), neurological and geriatric conditions (e.g., stroke and frailty-related syndromes), and cardiometabolic conditions. Primary progressive neurological disorders, including Parkinson’s disease, Alzheimer’s disease, and dementia, were excluded because their progressive disease trajectories, cognitive impairment, and disease-specific functional limitations may independently influence balance, mobility, and responses to exercise interventions. Therefore, these conditions were considered clinically distinct from non-progressive chronic conditions targeted in the present review.

Studies were excluded if: (1) participants were institutionalized (nursing home residents) or belonged to specific populations such as athletes; (2) participants had primary neurological disorders (Alzheimer’s disease, Parkinson’s disease, or dementia); (3) interventions did not primarily involve exercise therapy; (4) the study design was non-randomized (observational studies, quasi-experimental studies, reviews, or conference abstracts); or (5) insufficient data were available to calculate effect sizes (**Supplementary Table 1**).

### 2.2 Information sources and search strategy

A comprehensive literature search was conducted across multiple electronic databases, including PubMed, Embase, Web of Science, Cochrane Library and CINAHL, from database inception to 8 March 2026.

The search strategy combined controlled vocabulary (MeSH terms) and free-text terms related to “older adults,” “chronic conditions,” “exercise therapy,” “balance,” “functional performance,” and “falls.” Restrictive NOT terms (Alzheimer OR Parkinson OR dementia OR “nursing home” OR athlete) were used to exclude records primarily focusing on populations or conditions outside the predefined scope of this review and to improve the specificity of the search. We acknowledge that the use of restrictive NOT terms may potentially exclude otherwise eligible records when multiple populations or conditions are included in the same study. Reference lists of included studies and relevant reviews were also screened to identify additional eligible studies. The full search strategy is provided in **Supplementary Material** (**Supplementary Table 2**).

### 2.3 Data collection process and data extraction

Covidence, Endnote, and Excel applications were used to manage records throughout the review process (17). The processes of duplicate removal, title and abstract screening, and full-text review were documented using the PRISMA flow diagram (16).

References were imported into the Covidence system for duplicate removal. Two researchers (Beihai Ge and Renming Liu) independently screened titles and abstracts using inclusion and exclusion criteria. Any disagreements were resolved through discussion. If no agreement could be reached, a third researcher (Xianhui Liao) made the decision. Two reviewers independently extracted data using standardized data extraction forms. If data was insufficient or unclear, the researchers contacted the study authors to obtain missing information. Extracted information included: first author, publication year, language, country/region, sample characteristics (sample size, mean age and/or age range), intervention characteristics (exercise modality and dosage, including frequency, duration, and session time), control characteristics (active or inactive control), and assessment tools used. For continuous outcomes, post-intervention endpoint scores were extracted rather than change-from-baseline scores. When studies reported multiple post-intervention or follow-up assessments, the longest available follow-up time point after completion of the exercise intervention was selected for meta-analysis. This approach was adopted to evaluate the sustained effects of exercise therapy and avoid including multiple effect estimates from the same study. Different follow-up time points were not combined within the same analysis to minimize potential heterogeneity arising from variations in assessment timing. Any disagreements were resolved through discussion; if no agreement could be reached, a third researcher made the decision. For studies excluded at the full-text stage, the reasons for exclusion were recorded. For trials containing more than two intervention or control groups, only comparisons that meet the pre-defined inclusion criteria and are relevant to the review question are included in the quantitative meta-analysis. For example, Zhao et al. (38) included a Tai Chi group, a balance training group, and a no-treatment group, but only extracted and analyzed the comparison between the Tai Chi group and the no-treatment group. When standard deviations were not directly reported, they were calculated or converted from other available statistical information when possible. If the required information could not be obtained through calculation or conversion, the study authors were contacted to request the missing data. When outcome measures were reported in different units, the values were converted to a common unit before quantitative synthesis. If a reliable conversion could not be performed, the corresponding data were not included in the quantitative analysis. Outcomes were selected for quantitative synthesis according to the outcome domains predefined in the PICO(S) framework of the review. Follow-up information was recorded for each study, including whether follow-up assessments were reported and the corresponding follow-up time points.

### 2.4 Subgroup analyses

To explore potential sources of heterogeneity, subgroup analyses were conducted according to chronic condition categories and exercise modalities when sufficient data were available. Based on the underlying health conditions reported in the included studies, participants were categorized into three chronic condition groups: (1) musculoskeletal-related chronic conditions, including osteoporosis and sarcopenia; (2) neurological and geriatric conditions, including stroke, frailty, and cognitive impairment; and (3) cardiometabolic conditions, including hypertension.

In addition, subgroup analyses were performed according to exercise modalities. exercise therapy were classified into three categories: (1) Tai Chi-based exercise; (2) balance training; and (3) other exercise interventions, including resistance training, home-based exercise, physical activity, group exercise, and exercise park interventions. Subgroup analyses were considered exploratory and were conducted only for outcomes with sufficient numbers of studies available.

### 2.5 Quality assessment

Two reviewers (Jinsong Luo and Xiaofeng Cheng) assessed the methodological quality and internal validity of eligible trials at the study level using the Cochrane Risk of Bias 2.0 tool (RoB 2.0) (18). For each trial that met the inclusion criteria, we assessed the risk of bias in five areas: randomization process, deviation from the intended intervention, missing outcome data, outcome measurement, and outcome reporting. For each area, we evaluated the specific study using the signaling issues listed in RoB 2.0 and determined the risk of bias as high, problematic, or low. Disagreements were resolved through discussion between the two reviewers, and if necessary, a third reviewer (Deng Wang) was involved to reach a consensus.

### 2.6 Data synthesis

All meta-analyses were performed using STATA 18.0 (19). Meta-analyses were used when three or more studies reported the same balance function or fall-related indicators. For continuous outcomes, weighted mean differences (WMD) were calculated when the same measurement scale was used across studies, whereas standardized mean differences (SMD) (20, 21) For continuous outcomes, weighted mean differences (WMDs) were calculated when the same measurement scale was used across studies, whereas standardized mean differences (SMDs) (22). I2 values were calculated, and rated as follows: low, 0–25%; moderate, 25–50%; moderate-high, 50-75%; high, 75–100% (23, 24). A two-sided p value <0.05 was considered statistically significant. Funnel plots and Egger’s tests were used to assess potential small-study effects only when at least 10 studies were available for a given outcome (25). When fewer than 10 studies were available, publication bias could not be reliably assessed.

### 2.7 Certainty of evidence assessment

The certainty of evidence for the main outcomes was assessed using the Grading of Recommendations Assessment, Development and Evaluation (GRADE) approach. The quality of evidence was independently evaluated by two reviewers (Beihai Ge and Jinsong Luo) based on five domains: risk of bias, inconsistency, indirectness, imprecision, and publication bias. The certainty of evidence was classified as high, moderate, low, or very low. Any disagreements between the two reviewers were resolved through discussion and consensus. A summary of findings table was generated to present the certainty of evidence for the main outcomes.

## 3 Results

### 3.1 Study selection

A total of 17,713 records were initially identified through database searching, including PubMed (n = 4,571), Cochrane Library (n = 3,274), Embase (n = 4,372), CINAHL (n = 1,586), and Web of Science (n = 3,910). After duplicate removal, the remaining records were screened based on titles and abstracts. Full-text articles were subsequently assessed for eligibility according to the predefined inclusion and exclusion criteria. Studies were excluded for reasons including non-randomized study design, irrelevant interventions or outcomes, non-community-dwelling populations, and insufficient data for quantitative synthesis. Ultimately, 14 randomized controlled trials (RCTs) were included in the systematic review and meta-analysis (26–39). The detailed study selection process is illustrated in the PRISMA flow diagram (**Figure 1**).

**Figure 1:**
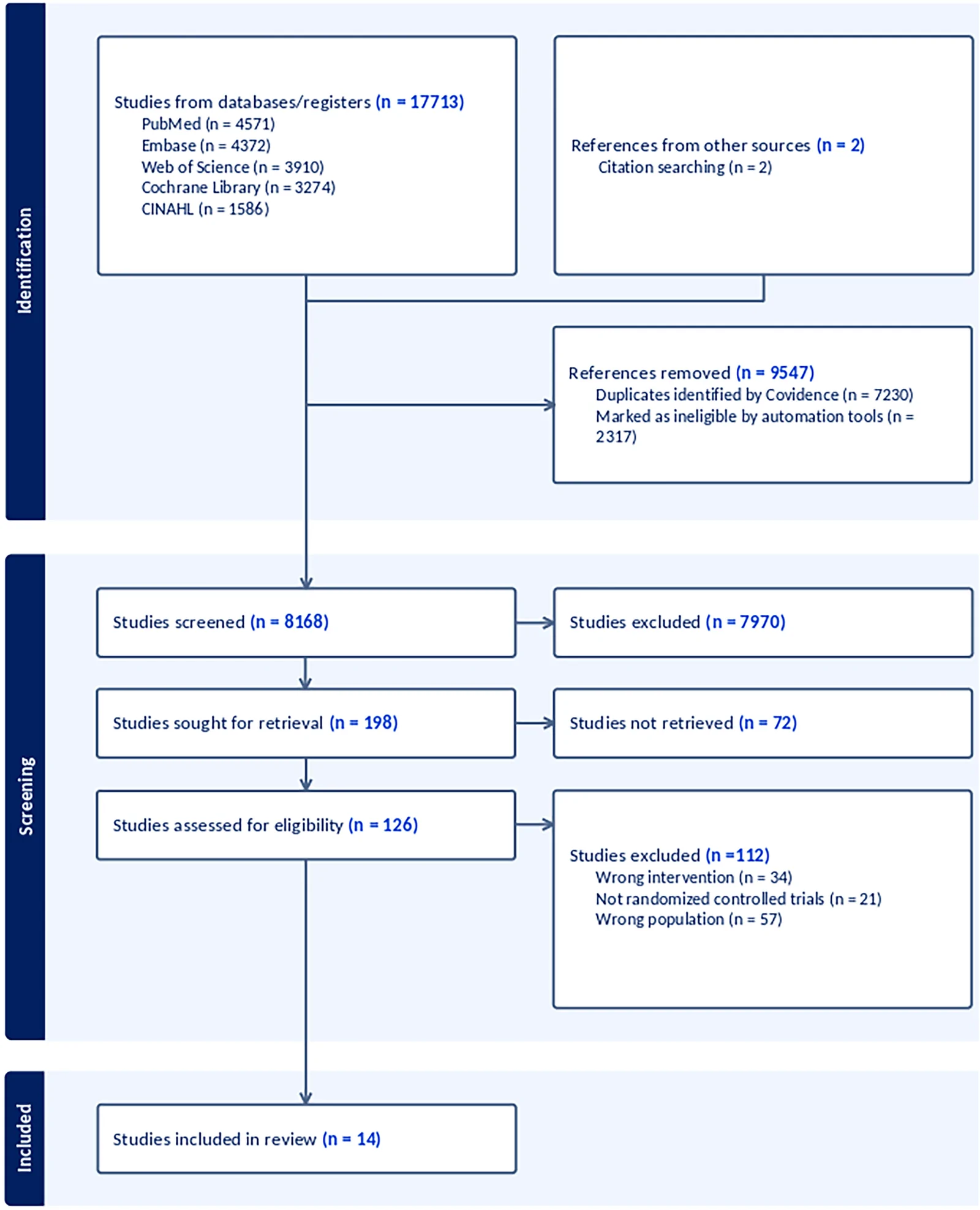
Flowchart of literature screening and inclusion for systematic review and meta-analysis.

### 3.2 Study characteristics

The 14 included studies comprised a total of 857 participants, with a mean age of 72.03 years. Female participants accounted for most of the study population (568 participants, 66.3%), whereas male participants represented 251 participants (29.3%). Sex information was not reported for 38 participants (4.4%) from one study (Niebauer et al., (31)). The studies included were conducted across diverse geographical regions. China contributed the largest number of studies (n = 5, 35.7%). The remaining studies were conducted in United States, Australia, Korea, Sweden, Austria, Hungary, Thailand, Iran, and Germany (n = 1 study each, 7.1%). All included articles were published in English (**Table 1**). Participants were community-dwelling older adults aged ≥60 years with chronic conditions. The included studies were categorized into three major condition groups: musculoskeletal-related chronic conditions, neurological and geriatric conditions, and cardiometabolic conditions. Exercise therapy varied across studies and were classified as Tai Chi-based exercise, balance training, and other exercise interventions, which included resistance training, home-based exercise, physical activity, group exercise, and exercise park interventions (**Table 2**). Considerable variability was observed in exercise dosage across the included studies. Intervention duration ranged from 8 to 48 weeks. Exercise frequency varied from once every two weeks to three sessions per week, although some studies reported the total number of sessions rather than weekly frequency. Session duration ranged from 30 to 120 minutes when reported. Detailed intervention characteristics are summarized in **Table 3**.

**Table 1:** General participant and study characteristics.

Characteristics		Number (%)	Mean
Total sample size		857 (100)	
Age, years			72.03
Sex			
Male participants, n (%)		251 (29.3)	
Female participants, n (%)		568 (66.3)	
No Report		38 (4.4) *	
Country			
China		5 (35.7)	
United States		1 (7.1)	
Brazil		1 (7.1)	
Australia		1 (7.1)	
Korea		1 (7.1)	
Sweden		1 (7.1)	
Austria		1 (7.1)	
Hungary		1 (7.1)	
Thailand		1 (7.1)	
Iran		1 (7.1)	
Germany		1 (7.1)	
Language			
English		14 (100)	

*Niebauer et al., (31) data on sex not reported.

General participant and study characteristics (n= 14).

**Table 2:** Characteristics of the included studies.

First author (year, language, location)	Sample characteristics				
	Sample size	Sex	Age	Disease	Intervention/control
Wu et al. ((35), English, United States)	13	Male/female: 2/11	80.5 ± 6.8	Cardiometabolic conditions	Tai Chi-based exercise
	10	Male/female: 2/8	79.1 ± 5.1		Usual care
Sales et al. ((32), English, Australia)	27	Male/female: 10/17	75.1 ± 7.95	Neurological and geriatric conditions	Other exercise interventions
	21	Male/female: 5/16	70.2 ± 8.20		Usual care
Yuen et al. ((36), English, China)	29	Male/female: 15/14	63.1 ± 10.6	Neurological and geriatric conditions	Tai Chi-based exercise
	29	Male/female: 14/15	62.0 ± 13.1		Usual care
Halvarsson et al. ((27), English, Sweden)	25	Male/female: 0/25	76 ± 66-87	Musculoskeletal-related chronic conditions	Balance training
	26	Male/female: 1/25	76 ± 68-85		Usual care
Niebauer et al. ((31), English, Austria)	20	Male/female: NR	74.6 ± 6.0	Musculoskeletal-related chronic conditions	Balance training
	18	Male/female: NR	78.9 ± 7.7		Usual care
Zhao et al. ((38), English, China)	20	Male/female: 6/14	70.2 ± 3.9	Musculoskeletal-related chronic conditions	Tai Chi-based exercise
	20	Male/female: 5/15	69.9 ± 3.3		Usual care
Tou et al. ((34), English, China)	29	Male/female: 5/24	72.9 ± 8.0	Neurological and geriatric conditions	Tai Chi-based exercise
	27	Male/female: 2/25	72.6 ± 5.7		Usual care
Miko et al. ((29), English, Hungary)	50	Male/female: 0/50	69.33 ± 4.56	Musculoskeletal-related chronic conditions	Balance training
	50	Male/female: 0/50	69.10 ± 5.30		Usual care
Zhang et al. ((37), English, China)	34	Male/female: 5/29	68.4 ± 4.9	Musculoskeletal-related chronic conditions	Other exercise interventions
	34	Male/female: 6/28	68.4 ± 4.6		Usual care
Huang et al. ((39), English, China)	27	Male/female: 17/10	69.70 ± 5.05	Musculoskeletal-related chronic conditions	Tai Chi-based exercise
	29	Male/female: 14/15	72.14 ± 4.79		Usual care
Sungkarat et al. ((33), English, Thailand)	33	Male/female: 2/31	68.3 ± 6.7	Neurological and geriatric conditions	Tai Chi-based exercise
	33	Male/female: 7/26	67.5 ± 7.3		Usual care
Lee et al. ((28), English, Korea)	28	Male/female: 17/11	80.39 ± 2.57	Musculoskeletal-related chronic conditions	Balance training
	29	Male/female: 14/15	79.10 ± 3.90		Usual care
Mortazavi et al. ((30), English, Iran)	27	Male/female: 13/14	67.20 ± 5.416	Neurological and geriatric conditions	Tai Chi-based exercise
	26	Male/female: 13/13	68.07 ± 5.232		Usual care
Freiberger et al. ((26), English, Germany)	63	Male/female: 33/30	76.1 ± 4.1	Neurological and geriatric conditions	Other exercise interventions
	80	Male/female: 43/37	76.8 ± 4.1		Usual care

NR, Not report.

**Table 3:** Dosage of intervening program.

First author (year, language, location)	Dosage			
	Intervention/control	Duration (weeks)	Frequency (per week)	Intensity (min)
Wu et al. ((35), English, United States)	Tai Chi-based exercise	12	3	60
	Usual care	NR	NR	NR
Sales et al. ((32), English, Australia)	Other exercise interventions	18	once every two weeks	120
	Usual care	NR	NR	NR
Yuen et al. ((36), English, China)	Tai Chi-based exercise	8	3	50
	Usual care	NR	NR	NR
Halvarsson et al. ((27), English, Sweden)	Balance training	12	3	45
	Usual care	NR	NR	NR
Niebauer et al. ((31), English, Austria)	Balance training	8	3	NR
	Usual care	NR	NR	NR
Zhao et al. ((38), English, China)	Tai Chi-based exercise	16	3	90
	Usual care	NR	NR	NR
Tou et al. ((34), English, China)	Tai Chi-based exercise	16	44 times in total	60
	Usual care	NR	NR	NR
Miko et al. ((29), English, Hungary)	Balance training	48	3	30
	Usual care	NR	NR	NR
Zhang et al. ((37), English, China)	Other exercise interventions	12	3	60
	Usual care	NR	NR	NR
Huang et al. ((39), English, China)	Tai Chi-based exercise	12	3	40
	Usual care	NR	NR	NR
Sungkarat et al. ((33), English, Thailand)	Tai Chi-based exercise	12	3	50
	Usual care	NR	NR	NR
Lee et al. ((28), English, Korea)	Balance training	8	1	50
	Usual care	NR	NR	NR
Mortazavi et al. ((30), English, Iran)	Tai Chi-based exercise	10	3	NR
	Usual care	NR	NR	NR
Freiberger et al. ((26), English, Germany)	Other exercise interventions	16	2	60
	Usual care	NR	NR	NR

NR, Not report.

Control conditions primarily consisted of usual care, health education, wait-list controls, or no structured exercise intervention. Reported outcome measures covered multiple domains, including balance, mobility, functional performance, and fall-related outcomes. Commonly assessed measures included the Timed Up and Go test (TUG), Berg Balance Scale (40), One-Leg Stand test (OLS), Functional Reach Test (FRT), sit-to-stand tests, gait performance measures, and fall-related psychological assessments such as fear of falling and Falls Efficacy Scale-International (FES-I). A detailed summary of outcome measures reported in the included studies is provided in **Table 4**.

**Table 4:** Outcome indicators, adverse events, and follow-ups included in the studies.

First author (year, language, location)	Outcome	Adverse events (Yes/No/No report)	Reasons for dropout	Follow-up (Yes/No/No report)
Wu et al. ((35), English, United States)	Postural Stability Test-overall, Timed Up and Go Test, Four Square Step Test, 10-Meter Walk Test	Yes	N = 3: Conflict of Schedule (n = 1) Change of Health Status (n = 2)	Yes
	Postural Stability Test-overall, Timed Up and Go Test, Four Square Step Test, 10-Meter Walk Test	No	/	Yes
Sales et al. ((32), English, Australia)	BOOMER - Total, Single Leg Stance,Knee Strength, Two Minute Walk, Timed Up and Go Fast, Sit to Stand, Gait Speed, Short FES-I, SF12 PCS, SF12 MCS	No report	/	No report
	BOOMER - Total, Single Leg Stance, Knee Strength, Two Minute Walk, Timed Up and Go Fast, Sit to Stand, Gait Speed, Short FES-I, SF12 PCS, SF12 MCS	No report	/	No report
Yuen et al. ((36), English, China)	Mini-BESTest, LOS-RT, LOS-EPE, SOT, FTSTT, TUG, FES-I, SS-QOL	Yes	N = 2: Dropout (n = 2)	Yes
	Mini-BESTest, LOS-RT, LOS-EPE, SOT, FTSTT, TUG, FES-I, SS-QOL	Yes	N = 2: Dropout (n = 2)	Yes
Halvarsson et al. ((27), English, Sweden)	FES-I, OLS, Fear of falling	Yes	N = 18: Lostto follow-up (n = 9), Due to illness (n = 6), Declining interest (n = 3)	Yes
	FES-I, OLS, Fear of falling	Yes	N = 10: Lostto follow-up (n = 5), Due toillness (n = 3), Declining interest (n = 1), Personaj reason (n = 1)	Yes
Niebauer et al. ((31), English, Austria)	Functional reach, POMA – balance score, POMA – gait score, POMA – total score, TUG, SPPB – sum balance, SPPB – sum gait speed, SPPB – sum chair test, SPPB – total score	No report	/	No report
	Functional reach, POMA – balance score, POMA – gait score, POMA – total score, TUG, SPPB – sum balance, SPPB – sum gait speed, SPPB – sum chair test, SPPB – total score	No report	/	No report
Zhao et al. ((38), English, China)	30-s Arm curl, 30-s Chair stand, Back scratch, Chair sit-and-reach, 8-ft up and go, 2-min step	Yes	N = 3: Dropped out (n = 3)	Yes
	30-s Arm curl, 30-s Chair stand, Back scratch, Chair sit-and-reach, 8-ft up and go, 2-min step	No	/	Yes
Tou et al. ((34), English, China)	KES, MQ, Falls efficacy, 6-m fast GS, TUG, 30-s sit-to-stand, EQ-5D index score, Frailty	Yes	N = 4: Dropped out (n = 4)	Yes
	KES, MQ, Falls efficacy, 6-m fast GS, TUG, 30-s sit-to-stand, EQ-5D index score, Frailty	Yes	N = 2: Dropped out (n = 2)	Yes
Miko et al. ((29), English, Hungary)	TUG	Yes	N = 1: Dropped out (n = 1)	Yes
	TUG	Yes	N = 2: Dropped out (n = 2)	Yes
Zhang et al. ((37), English, China)	FTSST, HGS, TUGT, BBS	Yes	N = 2: Moving out of the residence (n = 1), Refusal to continue (n = 1)	Yes
	FTSST, HGS, TUGT, BBS	Yes	N = 2: Unable to follow up regularly (n = 2)	Yes
Huang et al. ((39), English, China)	OSI	Yes	N = 3: Dropped out (n = 3)	Yes
	OSI	Yes	N = 1: Dropped out (n = 1)	Yes
Sungkarat et al. ((33), English, Thailand)	PPA fall risk index score,	Yes	N = 3: Dropped out (n = 3)	Yes
	PPA fall risk index score,	Yes	N = 4: Dropped out (n = 4)	Yes
Lee et al. ((28), English, Korea)	OLST, BBS, FRT, TUGT, FTSTS, MFES	Yes	N = 2: Dropped out (n = 2)	Yes
	OLST, BBS, FRT, TUGT, FTSTS, MFES	Yes	N = 1: Dropped out (n = 1)	Yes
Mortazavi et al. ((30), English, Iran)	falling and fear of falling	Yes	N = 3: Dropped out (n = 3)	Yes
	falling and fear of falling	Yes	N =4: Dropped out (n = 4)	Yes
Freiberger et al. ((26), English, Germany)	TUG, Walking speed, fast pace, s/6m	Yes	N = 7: Dropped out (n = 7)	Yes
	TUG, Walking speed, fast pace, s/6m	Yes	N = 17: Dropped out (n = 17)	Yes

TUG, Timed Up and Go Test; UWS, Usual Walking Speed; MWS, Maximum Walking Speed; ACT, Activity Confidence Test; FAB, Fullerton Advanced Balance Scale; LOS-RT, Limits of Stability–Reaction Time; LOS-EPE, Limits of Stability–Endpoint Excursion; SOT, Sensory Organization Test; FTSTT, Five Times Sit-to-Stand Test; FES-I, Falls Efficacy Scale–International; SS-QOL, Stroke-Specific Quality of Life Scale; UBT, Up and Balance Test; CST, Chair Stand Test; OLS, One-Leg Stance; POMA, Performance-Oriented Mobility Assessment; SPPB, Short Physical Performance Battery; KES, Knee Extension Strength; MQ, Muscle Quality; GS, Gait Speed; EQ-5D, EuroQol 5-Dimension Questionnaire; BBS, Berg Balance Scale; FTSST, Five Times Sit-to-Stand Test; HGS, Hand Grip Strength; TUGT, Timed Up and Go Test; OSI, Overall Stability Index; CSRT, Choice Stepping Reaction Time; SST, Simple Stepping Test; SDMT, Symbol Digit Modalities Test; TMT B-A, Trail Making Test Part B minus Part A; PPA, Physiological Profile Assessment; FRT, Functional Reach Test; DGI, Dynamic Gait Index; 10MWT, 10-Meter Walk Test; OLST, One-Leg Stance Test; MFES, Modified Falls Efficacy Scale; SF12 PCS, 12-Item Short Form Health Survey Physical Component Summary; SF12 MCS, 12-Item Short Form Health Survey Mental Component Summary; Mini-BESTest, Mini-Balance Evaluation Systems Test; FTSTS, Five Times Sit-to-Stand Test.

### 3.3 Effects of exercise therapy on balance and functional outcomes

Pooled analyses demonstrated that exercise therapy significantly improved multiple objective indicators of balance and functional performance among community-dwelling older adults with chronic conditions. Specifically, exercise therapy significantly enhanced TUG performance compared with control conditions (WMD = -0.76, 95% CI: -1.50 to -0.02, I^2^ = 38.3%, p = 0.04), indicating improved functional mobility and dynamic balance.

In addition, exercise therapy significantly improved OLS performance (WMD = 5.45, 95% CI: 1.45 to 9.44, I^2^ = 62.2%, p = 0.008), although moderate heterogeneity was observed, potentially attributable to variations in intervention modalities and participant characteristics across studies.

Overall, the findings suggest that exercise therapy confers robust and consistent benefits on objective balance and functional performance outcomes in community-dwelling older adults with chronic conditions. These findings indicate that exercise therapy may provide meaningful improvements in objective measures of functional mobility, balance capacity, and postural control, including TUG, and OLS outcomes (**Figures 2**, **3**; **Supplementary Table 3**).

**Figure 2:**
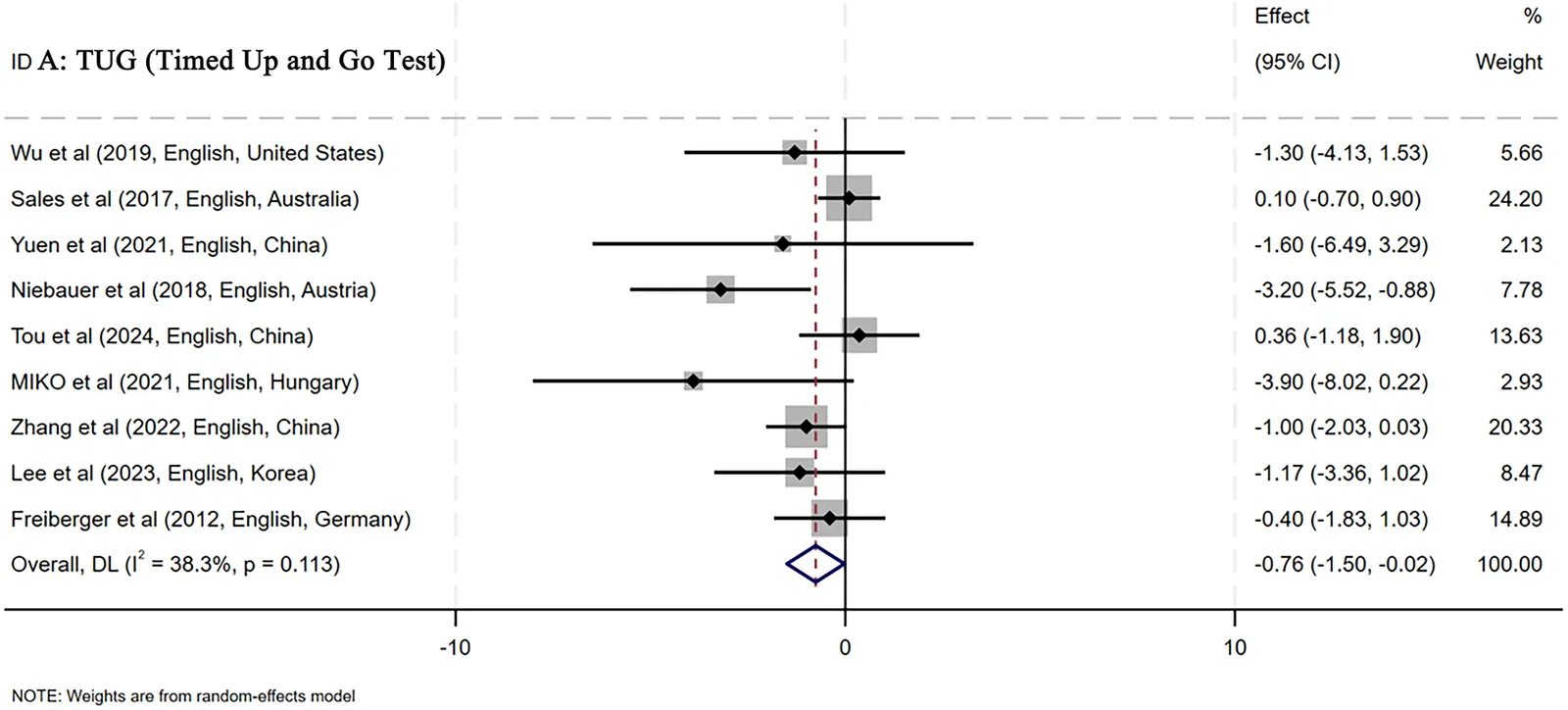
Effects of exercise therapy on balance and functional outcomes. **(A)** Timed Up and Go (TUG) test.

**Figure 3:**
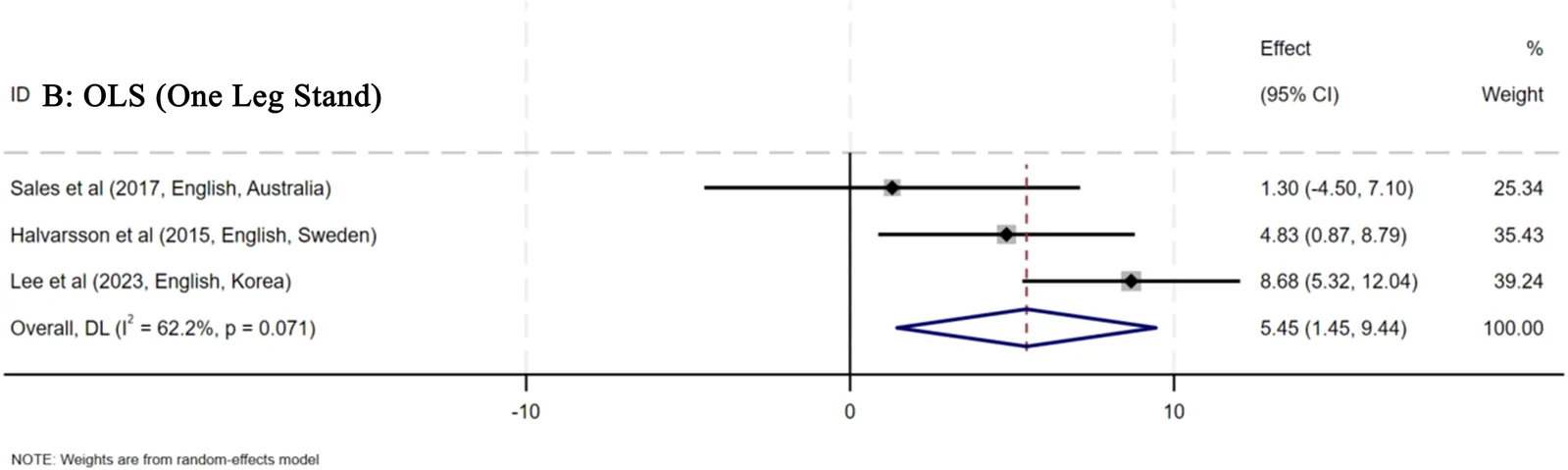
Effects of exercise therapy on balance and functional outcomes. **(B)** One-Leg Stand (OLS) test.

### 3.4 Subgroup analyses according to chronic condition categories and exercise modalities

To explore potential sources of heterogeneity, exploratory subgroup analyses were conducted according to chronic condition categories and exercise modalities when sufficient data were available. For chronic condition categories, included studies were classified into three groups: (1) musculoskeletal-related chronic conditions, including osteoporosis and sarcopenia; (2) neurological and geriatric conditions, including stroke, frailty, and cognitive impairment; and (3) cardiometabolic conditions, including hypertension. No significant differences between exercise modality subgroups were identified.

For TUG performance, exercise interventions were associated with improved functional mobility in the musculoskeletal-related chronic condition subgroup (WMD = −1.71, 95% CI: −2.93 to −0.49, I^2^ = 31%, P = 0.006). In the neurological and geriatric condition subgroup, no statistically significant effect was observed (WMD = 0.02, 95% CI: −0.61 to 0.65, I^2^ = 0%, P = 0.96). The cardiometabolic subgroup included only one study and showed no statistically significant effect (WMD = −1.30, 95% CI: −4.13 to 1.53, P = 0.37). The formal test for subgroup differences indicated a statistically significant difference across the three chronic condition categories (χ^2^ = 6.54, df = 2, P = 0.038).

Regarding exercise modalities, interventions were categorized into three groups: (1) Tai Chi-based exercise; (2) balance training; and (3) other exercise interventions, including resistance training, home-based exercise, physical activity, group exercise, and exercise park interventions. For TUG performance, the Tai Chi-based exercise subgroup showed no statistically significant effect (WMD = −0.13, 95% CI: −1.44 to 1.17, I^2^ = 0%, P = 0.84). Balance training was associated with a statistically significant improvement in TUG performance (WMD = −2.38, 95% CI: −3.95 to −0.81, I^2^ = 8%, P = 0.003), whereas other exercise interventions showed no statistically significant effect (WMD = −0.37, 95% CI: −1.07 to 0.34, I^2^ = 27%, P = 0.31). However, the formal test for subgroup differences did not indicate a statistically significant difference among the three exercise modality subgroups (χ^2^ = 5.88, df = 2, P = 0.053) (**Supplementary Tables 4**, **5**).

Overall, these subgroup findings should be interpreted as exploratory and hypothesis-generating, particularly because some subgroups contained relatively few studies.

### 3.5 Risk of bias

The risk of bias was assessed using the RoB 2 tool at the outcome. Across the 14 included studies, no study was judged to have an overall low risk of bias; all 14 studies were assessed as having some concerns. For deviations from intended interventions, five studies were judged to have a low risk of bias, whereas nine studies had some concerns. For missing outcome data, three studies were judged to have a low risk of bias and 11 had some concerns. For measurement of the outcome, five studies were judged to have a low risk of bias, whereas nine had some concerns. For selection of the reported result, six studies were judged to have a low risk of bias and eight had some concerns. Overall, the included studies were predominantly assessed as having some concerns across the RoB 2 domains. The detailed risk-of-bias assessments are presented in **Supplementary Table 6** and **Figure 4**.

**Figure 4:**
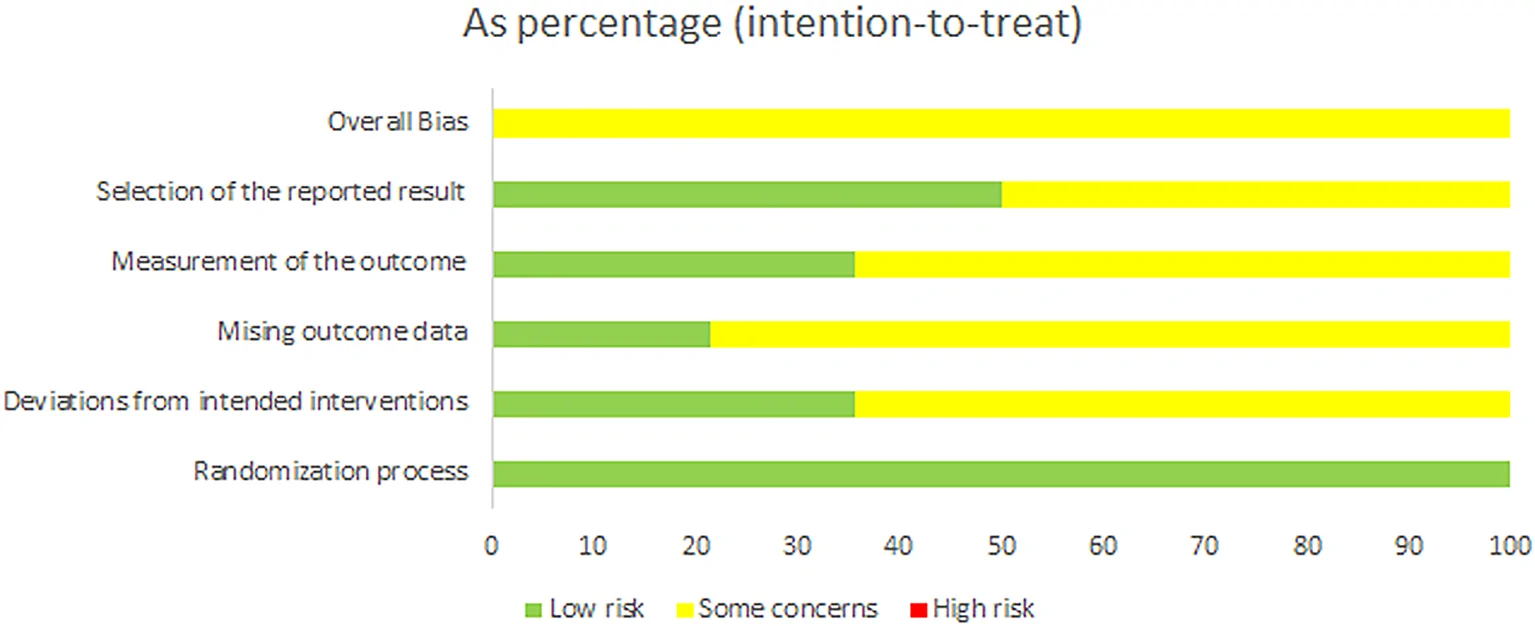
Risk of bias graph.

### 3.6 Publication bias

Publication bias could not be reliably assessed for the included outcomes because the number of studies was insufficient for reliable interpretation of funnel plot asymmetry. The TUG analysis included nine studies, while the OLS analysis included only three studies. Therefore, funnel plots and Egger’s tests were not considered sufficiently reliable for assessing publication bias for these outcomes (**Figure 5**).

**Figure 5:**
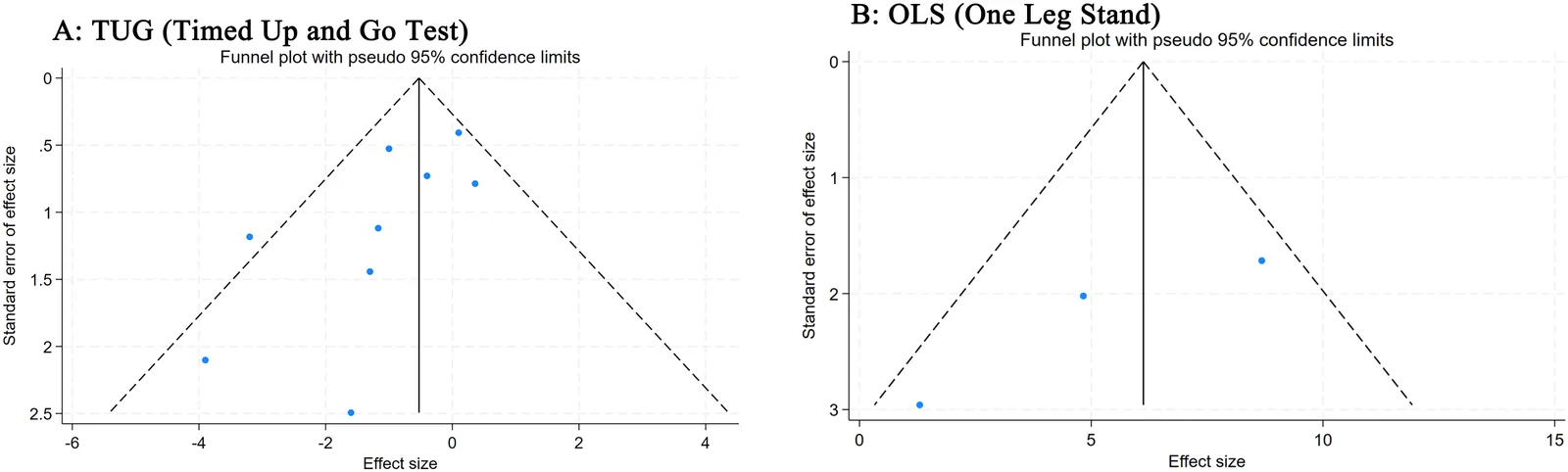
Funnel plots for publication bias assessment. **(A)** Timed Up and Go (TUG) test; **(B)** One-Leg Stand (OLS) test.

### 3.7 Certainty of evidence

The certainty of evidence for the main outcomes was assessed using the GRADE approach. The certainty of evidence ranged from low to moderate. For the TUG test, the certainty of evidence was rated as low, with downgrading by one level for risk of bias and one level for inconsistency. The downgrading for risk of bias reflected some concerns or high risk of bias across the included studies, while inconsistency was considered serious because of differences in the magnitude and precision of the study estimates. For the OLS test, the certainty of evidence was rated as moderate and was downgraded by one level for risk of bias. Although substantial statistical heterogeneity was observed for OLS (I^2^ = 62.2%), the direction of effects was generally consistent across studies; therefore, the evidence was not downgraded for inconsistency. No downgrading was applied for indirectness or imprecision for either outcome. A summary of the GRADE assessment is presented in **Table 5**.

**Table 5:** Certainty of evidence assessment using the GRADE approach.

Certainty assessment							№ of patients		Effect		Certainty	Importance
№ of studies	Study design	Risk of bias	Inconsistency	Indirectness	Imprecision	Other considerations	[intervention]	[comparison]	Relative (95% CI)	Absolute (95% CI)		
TUG (Timed Up and Go Test)												
9	randomised trials	serious^a^	serious^b^	not serious	not serious	none	280	265	–	MD 0.76 lower (1.5 lower to 0.02 lower)	⨁⨁◯◯Low^a^,^b^	CRITICAL
OLS (One Leg Stand)												
3	randomised trials	serious^a^	not serious^c^	not serious	not serious	none	80	76	–	MD 5.45 higher (1.45 higher to 9.44 higher)	⨁⨁⨁◯ Moderate^a^^c^	CRITICAL

CI, confidence interval; MD, mean difference.

^a^ Some concerns or high risk of bias in included studies.

^b^ The study results show inconsistencies.

^c^ The results of the study show a high degree of heterogeneity.

## 4 Discussion

This systematic review and meta-analysis synthesized the current evidence regarding the effects of exercise therapy on balance, functional performance, and fall-related outcomes in community-dwelling older adults with chronic diseases. The pooled findings demonstrated that exercise therapy significantly improved objective indicators of balance and functional mobility. Specifically, significant improvements were observed in OLS and TUG outcomes, suggesting beneficial effects of exercise therapy on postural control and mobility among community-dwelling older adults with chronic conditions.

The magnitude of these improvements should be interpreted considering their clinical relevance. Although statistically significant benefits were observed, clinically meaningful thresholds are not consistently established for all balance outcomes. Therefore, the findings indicate potential functional benefits of exercise therapy but should be interpreted cautiously regarding their direct translation into meaningful improvements in daily activities or fall prevention. For TUG, the pooled effect was relatively small (WMD = −0.76 s, 95% CI: −1.50 to −0.02). Although this change was statistically significant, its clinical importance remains uncertain. Given the diversity of chronic conditions and baseline functional status across the included studies, a single clinically important threshold may not be directly applicable to all populations represented in this review. Therefore, the statistical significance of the TUG result should not necessarily be interpreted as evidence of a clinically meaningful improvement in daily functioning.

The beneficial effects observed in physical performance outcomes are likely attributable to physiological adaptations induced by exercise training. Balance and multicomponent exercise therapy may improve neuromuscular coordination, proprioceptive function, postural stability, and lower-extremity muscle strength, all of which are critical determinants of mobility and fall prevention in older adults (41, 42). In addition, resistance and functional training may enhance muscle power and motor efficiency, thereby improving dynamic balance and mobility, as reflected by better TUG performance (43). Collectively, these findings suggest that exercise therapy may exert beneficial effects through multiple biomechanical and neuromuscular mechanisms that help attenuate functional decline in older adults with chronic diseases.

Although the overall effects were favorable, heterogeneity was observed for some outcomes, particularly TUG and OLS. Several factors may contribute to this variability, including differences in chronic condition profiles, baseline functional status, exercise modalities, intervention dosage, and outcome assessment methods (44). The included studies represented diverse chronic conditions, including musculoskeletal-related chronic conditions, neurological and geriatric conditions, and cardiometabolic conditions, which may influence responsiveness to exercise therapy (45). Therefore, pooled estimates should be interpreted as average effects across heterogeneous populations rather than evidence supporting a single optimal exercise approach. The findings of the present study are generally consistent with previous evidence demonstrating that exercise therapy improves balance and functional performance in older adults (46, 47). However, this study extends the current literature in several important ways. First, unlike many previous reviews focusing on healthy older adults or individuals with a single disease condition, the present analysis specifically targeted community-dwelling older adults with chronic diseases, a population at particularly high risk of mobility limitation and falls. Second, this review incorporated multiple objective balance and functional performance outcomes, thereby providing a more comprehensive evaluation of intervention effectiveness. Furthermore, subgroup analyses based on chronic conditions and exercise modalities were conducted for TUG outcomes, suggesting that intervention effects may vary according to population characteristics and exercise approaches.

The findings of this study also have important clinical implications. Exercise therapy appears to be an effective non-pharmacological strategy for improving balance and functional mobility in community-dwelling older adults with chronic diseases. In particular, multicomponent exercise therapy integrating balance, resistance, and functional training may provide greater benefits (48). Balance-focused interventions demonstrated greater improvements in TUG performance in subgroup analyses, although the limited number of studies prevents definitive conclusions regarding optimal exercise modality (49). Importantly, the subgroup analyses were exploratory, and the formal test for subgroup differences did not demonstrate a statistically significant difference between exercise modalities (χ^2^ = 5.88, df = 2, P = 0.053). Future intervention programs may need to consider disease-specific characteristics and individualized exercise prescriptions.

Several limitations should be considered when interpreting these findings. First, substantial clinical heterogeneity existed among included studies regarding chronic condition categories, participant characteristics, baseline functional status, and disease severity, which may have influenced pooled estimates. Second, considerable variability was observed in exercise interventions, including differences in exercise modality, intervention duration (6–48 weeks), frequency (1–5 sessions/week), and session duration (30–120 minutes), which may have contributed to heterogeneity among pooled outcomes. The diversity in exercise prescriptions may partially explain variations in intervention effects, and the pooled estimates should therefore be interpreted cautiously rather than representing the effect of a standardized exercise protocol. Due to insufficient evidence, the independent effects of different exercise dosages could not be determined, limiting the ability to establish an optimal exercise prescription. Third, several included studies had relatively short intervention or follow-up periods, restricting evaluation of the long-term sustainability of exercise effects. Fourth, only English-language publications were included, which may have introduced potential language bias. Fifth, subgroup analyses could only be performed for outcomes with sufficient numbers of studies, while further exploration was not feasible for outcomes with limited available evidence. Additionally, although some included studies reported actual fall outcomes, including falls and injurious falls, these outcomes were not quantitatively synthesized because of substantial variability in fall definitions, assessment periods, and reporting methods, as well as insufficient comparable data across studies (50). Therefore, the effect of exercise therapy on reducing actual fall events remains uncertain. Similarly, fear of falling was not quantitatively synthesized, and therefore the effects of exercise therapy on fear of falling could not be established in this review. Overall, exercise interventions may improve selected measures of balance and functional mobility, although the certainty and clinical importance of these effects remain uncertain. Effects on falls and fear of falling were not established in this review. Furthermore, although significant improvements were observed in several functional outcomes, the lack of universally accepted minimal clinically important differences for some measures limited the interpretation of whether these changes translate into meaningful improvements in daily functioning. Finally, although the certainty of evidence was assessed using the GRADE approach, the evidence quality remained limited for some outcomes due to concerns regarding risk of bias and inconsistency.

## 5 Conclusion

This systematic review and meta-analysis demonstrate that exercise therapy improves objective indicators of balance and functional performance in community-dwelling older adults with chronic diseases. Significant benefits were observed in functional mobility outcomes, supporting exercise therapy as an important non-pharmacological intervention for maintaining functional capacity in this population.

However, the certainty of evidence varied across outcomes, and current evidence remains insufficient to determine the optimal exercise prescription for specific chronic condition populations. Although exercise interventions may improve selected measures of balance and functional mobility, the clinical importance of these effects remains uncertain. Furthermore, the effects of exercise therapy on falls and fear of falling were not established in this review. Future high-quality randomized controlled trials with standardized exercise protocols, multidimensional outcome assessments, and longer follow-up periods are needed to further identify the most effective exercise strategies for improving functional outcomes in older adults with chronic diseases.

## Data Availability

The original contributions presented in the study are included in the article/**Supplementary Material**. Further inquiries can be directed to the corresponding author.
