# Supplementary material for: Effects of exercise therapy on balance, functional performance, and fall-related outcomes in community-dwelling older adults with chronic conditions: a systematic review and meta-analysis
Source: Front Immunol. 2026 Oct 20;17:1918701. doi: 10.3389/fimmu.2026.1918701 (PMC13727617; doi:10.3389/fimmu.2026.1918701)
Supplement: Supplementary file 1 [file SupplementaryFile1.docx]

**Supplementary Table 1 The PICOs details**

| **Participant** | **Intervention** | **Control** | **Outcomes** | **Studies** |
| --- | --- | --- | --- | --- |
| Community-dwelling older adults aged ≥60 years with chronic conditions, including cardiovascular, metabolic, musculoskeletal, neurological, or other chronic diseases. Participants residing in nursing homes or long-term care institutions were excluded. | Exercise therapy interventions, including but not limited to balance training, resistance training, aerobic exercise, Tai Chi, multicomponent exercise therapy, functional training, and physical activity interventions aimed at improving balance, mobility, functional performance, or fall-related outcomes. | Usual care, health education, wait-list control, no intervention, or other non-exercise control conditions. | Studies reporting outcomes related to balance, mobility, functional performance, physical function, or fall-related outcomes were included. Both objective functional measures and subjective fall-related outcomes were considered. | Randomized controlled trials (RCTs) published in English. |
