# Supplementary material for: Effects of exercise therapy on balance, functional performance, and fall-related outcomes in community-dwelling older adults with chronic conditions: a systematic review and meta-analysis
Source: Front Immunol. 2026 Oct 20;17:1918701. doi: 10.3389/fimmu.2026.1918701 (PMC13727617; doi:10.3389/fimmu.2026.1918701)
Supplement: Supplementary file 2 [file SupplementaryFile2.docx]

**Supplementary Table 2 search strategy**

8 March 2026

PubMed: 4571

Cochrane Library: 3274

Embase: 4372

CINAHL: 1586

WEB OF SCIENCE: 3910

PubMed

#1: ("Accidental Falls"[Mesh] OR fall* OR falling)

#2: ("Exercise"[Mesh] OR "Exercise Therapy"[Mesh] OR exercise* OR training OR "physical activity" OR "tai chi" OR balance training)

#3: ("Aged"[Mesh] OR elderly OR older adult* OR older people OR senior*)

#4: (randomized controlled trial[pt] OR randomized[tiab] OR randomised[tiab] OR randomly[tiab] OR trial[tiab])

#5: #1 AND #2 AND #3 AND #4

#6: (Alzheimer* OR Parkinson* OR dementia OR "nursing home*" OR athlete*)

#7: (#5) NOT (#6)

**Cochrane Library**

#1 MeSH descriptor: [Accidental Falls] explode all trees
#2 fall* OR falling:ti,ab,kw
#3 #1 OR #2
#4 MeSH descriptor: [Exercise] explode all trees
#5 MeSH descriptor: [Exercise Therapy] explode all trees
#6 exercise* OR training OR "physical activity" OR "tai chi" OR "balance training":ti,ab,kw
#7 #4 OR #5 OR #6
#8 MeSH descriptor: [Aged] explode all trees
#9 elderly OR "older adult*" OR "older people" OR senior*:ti,ab,kw
#10 #8 OR #9
#11 randomized controlled trial:pt
#12 randomized OR randomised OR randomly OR trial:ti,ab,kw
#13 #11 OR #12
#14 #3 AND #7 AND #10 AND #13
#15 Alzheimer* OR Parkinson* OR dementia OR "nursing home*" OR athlete*:ti,ab,kw
#16 #14 NOT #15

Embase

#1 'accidental fall'/exp
#2 fall*:ti,ab,kw OR falling:ti,ab,kw
#3 #1 OR #2

#4 'exercise'/exp
#5 'exercise therapy'/exp
#6 exercise*:ti,ab,kw OR training:ti,ab,kw OR 'physical activity':ti,ab,kw OR 'tai chi':ti,ab,kw OR 'balance training':ti,ab,kw
#7 #4 OR #5 OR #6

#8 'aged'/exp
#9 elderly:ti,ab,kw OR 'older adult*':ti,ab,kw OR 'older people':ti,ab,kw OR senior*:ti,ab,kw
#10 #8 OR #9

#11 'randomized controlled trial'/exp
#12 random*:ti,ab,kw OR trial:ti,ab,kw
#13 #11 OR #12

#14 #3 AND #7 AND #10 AND #13

#15 Alzheimer*:ti,ab,kw OR Parkinson*:ti,ab,kw OR dementia:ti,ab,kw OR 'nursing home*':ti,ab,kw OR athlete*:ti,ab,kw

#16 #14 NOT #15

CINAHL

S1 (MH "Accidental Falls+")

S2 TI fall* OR AB fall* OR TI falling OR AB falling

S3 S1 OR S2

S4 (MH "Exercise+")

S5 (MH "Exercise Therapy+")

S6 TI exercise* OR AB exercise* OR TI training OR AB training OR TI "physical activity" OR AB "physical activity" OR TI "tai chi" OR AB "tai chi" OR TI "balance training" OR AB "balance training"

S7 S4 OR S5 OR S6

S8 (MH "Aged+")

S9 TI elderly OR AB elderly OR TI "older adult*" OR AB "older adult*" OR TI "older people" OR AB "older people" OR TI senior* OR AB senior*

S10 S8 OR S9

S11 TI random* OR AB random* OR TI trial OR AB trial

S12 S3 AND S7 AND S10 AND S11

S13 TI Alzheimer* OR AB Alzheimer* OR TI Parkinson* OR AB Parkinson* OR TI dementia OR AB dementia OR TI "nursing home*" OR AB "nursing home*" OR TI athlete* OR AB athlete*

S14 S12 NOT S13

WEB OF SCIENCE

TS=(fall* OR falling)

AND

TS=(exercise* OR training OR "physical activity" OR "tai chi" OR "balance training")

AND

TS=(elderly OR "older adult*" OR "older people" OR senior*)

AND

TS=(random* OR trial)

NOT

TS=(Alzheimer* OR Parkinson* OR dementia OR "nursing home*" OR athlete*)
