# Supplementary material for: Effects of exercise therapy on balance, functional performance, and fall-related outcomes in community-dwelling older adults with chronic conditions: a systematic review and meta-analysis
Source: Front Immunol. 2026 Oct 20;17:1918701. doi: 10.3389/fimmu.2026.1918701 (PMC13727617; doi:10.3389/fimmu.2026.1918701)
Supplement: Supplementary file 3 [file SupplementaryFile3.docx]

| Outcomes (Measurements) | WMD 95% CI, I2 | P value |
| --- | --- | --- |
| TUG (Timed Up and Go Test) | -0.76 [-1.50 to -0.02], I² = 38.3% | P=0.044* |
| OLS (One Leg Stand) | 5.45 [1.45 to 9.44], I² = 62.2% | P=0.008** |

**Supplementary Table 3 Overall effects**

**Note**: Difference *: p ＜ 0.05；**: p ＜ 0.01
