# Supplementary material for: Effects of exercise therapy on balance, functional performance, and fall-related outcomes in community-dwelling older adults with chronic conditions: a systematic review and meta-analysis
Source: Front Immunol. 2026 Oct 20;17:1918701. doi: 10.3389/fimmu.2026.1918701 (PMC13727617; doi:10.3389/fimmu.2026.1918701)
Supplement: Supplementary file 4 [file SupplementaryFile4.docx]

**Supplementary Table 4 Subgroup analysis by disease related group**

| **Outcome** | **Subgroup** | **studies** | **95% CI, I2** | **P value** |
| --- | --- | --- | --- | --- |
| TUG (Timed Up and Go Test) | Musculoskeletal-related chronic conditions | 4 | -1.71[-2.93 to -0.49], I² = 31% | 0.006* |
|  | Neurological and geriatric conditions | 4 | 0.02[-0.61 to 0.65], I² = 0% | 0.96 |
|  | Cardiometabolic conditions | 1 | -1.30[-4.13 to 1.53], I² = Not applicable | 0.37 |

Note: Difference *: p ＜ 0.05; **: p ＜ 0.01
