# Supplementary material for: Effects of exercise therapy on balance, functional performance, and fall-related outcomes in community-dwelling older adults with chronic conditions: a systematic review and meta-analysis
Source: Front Immunol. 2026 Oct 20;17:1918701. doi: 10.3389/fimmu.2026.1918701 (PMC13727617; doi:10.3389/fimmu.2026.1918701)
Supplement: Supplementary file 5 [file SupplementaryFile5.docx]

**Supplementary Table 5 Subgroup analysis by intervention group**

| **Outcome** | **Subgroup** | **studies** | **95% CI, I2** | **P value** |
| --- | --- | --- | --- | --- |
| TUG (Timed Stand-Up-Walk) | Balance training | 3 | -2.38[-3.95 to -0.81], I² = 8% | 0.003* |
|  | Tai Chi-based exercise | 3 | -0.13[-1.44 to 1.17], I² = 0% | 0.84 |
|  | Other exercise interventions | 3 | -0.37[-1.07 to 0.34], I² = 27% | 0.31 |

Note: Difference *: p ＜ 0.05; **: p ＜ 0.01
