# Supplementary material for: Effects of exercise therapy on balance, functional performance, and fall-related outcomes in community-dwelling older adults with chronic conditions: a systematic review and meta-analysis
Source: Front Immunol. 2026 Oct 20;17:1918701. doi: 10.3389/fimmu.2026.1918701 (PMC13727617; doi:10.3389/fimmu.2026.1918701)
Supplement: Supplementary file 6 [file SupplementaryFile6.docx]

**Supplementary Table 6 Risk of bias**

| **Unique ID** | **Study ID** | **Deviations from intended interventions** | **Missing outcome data** | **Measurement of the outcome** | **Selection of the reported result** | **Overall Bias** |
| --- | --- | --- | --- | --- | --- | --- |
| A1 | Wu et al (2021, English, United States) | Some concerns | Some concerns | Some concerns | Low | Some concerns |
| A2 | Sales et al (2017, English, Australia) | Some concerns | Low | Some concerns | Some concerns | Some concerns |
| A3 | Yuen et al (2021, English, China) | Low | Some concerns | Low | Low | Some concerns |
| A4 | Halvarsson et al (2015, English, Sweden) | Low | Some concerns | Some concerns | Low | Some concerns |
| A5 | Niebauer et al (2018, English, Austria) | Some concerns | Low | Some concerns | Some concerns | Some concerns |
| A6 | Zhao et al (2017, English, China) | Low | Low | Some concerns | Some concerns | Some concerns |
| A7 | Tou et al (2024, English, China) | Low | Some concerns | Low | Low | Some concerns |
| A8 | MIKO et al (2021, English, Hungary) | Some concerns | Some concerns | Some concerns | Some concerns | Some concerns |
| A9 | Zhang et al (2022, English, China) | Some concerns | Some concerns | Some concerns | Low | Some concerns |
| A10 | Huang et al (2023, English, China) | Some concerns | Some concerns | Low | Low | Some concerns |
| A11 | Sungkarat et al (2017, English, Thailand) | Some concerns | Some concerns | Low | Some concerns | Some concerns |
| A12 | Lee et al (2023, English, Korea) | Some concerns | Some concerns | Low | Low | Some concerns |
| A13 | Mortazavi et al (2018, English, Iran) | Some concerns | Some concerns | Some concerns | Some concerns | Some concerns |
| A14 | Freiberger et al (2012, English, Germany) | Low | Some concerns | Some concerns | Some concerns | Some concerns |
